# Draft Genome Sequences of Five Fungal Strains Isolated from Kefir

**DOI:** 10.1128/MRA.00195-21

**Published:** 2021-05-27

**Authors:** Simonas Marcišauskas, Yongkyu Kim, Sonja Blasche, Kiran R. Patil, Boyang Ji, Jens Nielsen

**Affiliations:** a Department of Biology and Biological Engineering, Chalmers University of Technology, Gothenburg, Sweden; b European Molecular Biology Laboratory, Heidelberg, Germany; c BioInnovation Institute, Copenhagen, Denmark; Vanderbilt University

## Abstract

We present the annotated draft genome sequences of five fungal strains isolated from kefir grains. These isolates included three ascomycetous (Candida californica, Kazachstania exigua, and Kazachstania unispora) and one basidiomycetous (Rhodotorula mucilaginosa) species. The results revealed a detailed overview of the metabolic features of kefir fungi that will be potentially useful in biotechnological applications.

## ANNOUNCEMENT

Kefir is fermented milk traditionally produced by a specific symbiotic culture of bacteria and fungi. Also known as kefir grains, this culture usually consists of 40 to 50 different species, including lactic acid bacteria, acetic acid bacteria, and yeasts (1). The ascomycetous yeast Kluyveromyces marxianus was previously identified in kefir grains (2), but little is known about other cooccurring fungi. Here, we report the annotated whole-genome sequences of the ascomycetous yeasts Candida californica, Kazachstania exigua, and Kazachstania unispora and the basidiomycetous fungus Rhodotorula mucilaginosa, isolated from kefir grains collected from private sources. These kefir grain cultures were collected in Germany (Ger04, *C. californica* and *K. unispora*; Ger06/OG2, K. exigua) and South Korea (Kefir Korea, *R. mucilaginosa*). *C. californica* SB-48 (referring internal stock identifier) was isolated from ground kefir grains and plated in serial dilutions onto yeast extract-peptone-dextrose-adenine (YPDA) medium. *C. californica* SB-116 was isolated and plated in serial dilutions onto Sabouraud dextrose (SD) medium. K. exigua SB-178 was isolated and plated in serial dilutions onto M17 medium supplemented with glucose. *K. unispora* SB-162 was isolated and plated in serial dilutions on de Man, Rogosa, and Sharpe (MRS) agar-milk agar (1/1 mix of MRS agar and 3.5% ultrahigh-temperature processing [UHT] milk). *R. mucilaginosa* SB-353 was isolated and plated in serial dilutions onto tomato juice agar (TJA). All isolates were grown in their corresponding medium for up to 5 days at 30°C. Isolates were identified by internal transcribed spaced (ITS) DNA amplification PCR using the primers S-D-Bact-0515-a-S-16 (GTGCCAGCMGCNGCGG) and S-*-Univ-1392-a-A-15 (ACGGGCGGTGTGTRC) (3) and subsequent Sanger sequencing of the amplified region. ITS sequences were taxonomically assigned using an open-reference method. The kefir-isolated yeast was used as the reference, and subsequent naive Bayesian classification was performed using UNITE (4). Strains were deposited and are available in the Leibniz Institute DSMZ collection of microorganisms under the same strain names.

The genomic DNA extraction was performed using a two-step approach combining enzymatic digestion with lysozyme, followed by bead beating with 0.3-g glass beads. The supernatant was then digested with proteinase K and applied to phenol-chloroform extraction and DNA precipitation, as described in references 5 and 6. DNA was then prepared for sequencing using a Nextera DNA library preparation kit (Illumina) and sequenced on an Illumina HiSeq 2000 instrument to get 100-bp paired-end reads with the insert size ranging between 250 bp and 300 bp. The quality of reads was checked with FastQC v0.11.9 (7), while Trimmomatic v0.36 (8) was used to adapter and quality trim the reads (with the following parameter settings: ILLUMINACLIP:TruSeq2-PE.fa:2:30:10 LEADING:3 TRAILING:3 SLIDINGWINDOW:4:15 MINLEN:36). A separate removal step for other contaminants was not performed. The resulting reads were assembled with ABySS v2.02 (9) and SPAdes v3.9.0 (10). No correction steps were performed for the ABySS assemblies; however, mismatches and short indels were corrected for the SPAdes assemblies (enabled with the --careful flag). The obtained genome assemblies based on different parameters were evaluated based on contiguity and completeness with single-copy orthologs using the QUAST v4.1 (11) and BUSCO v3 (12) tools, respectively. The lineage data sets in the benchmarking universal single-copy ortholog (BUSCO) analysis were saccharomycetes_odb9 (*C. californica*, K. exigua, and *K. unispora*) and basidiomycota_odb9 (*R. mucilaginosa*). The best genome assemblies were obtained with ABySS with k-mer length values (parameter k) set to 45 for K. exigua and 67 for *R. mucilaginosa*. Regarding the other three strains, SPAdes with default settings and the --careful flag produced the best assemblies. The contigs shorter than 500 bp were discarded.

Assemblies were annotated for repeat regions and soft masked with the RepeatModeler v1.0.11 (13) and RepeatMasker v4.0.7 (14) tools. The protein-encoding sequences (CDSs) and tRNAs were predicted with the funannotate predict function in funannotate v1.5.3 (15). The predicted genes were functionally annotated based on their protein sequences using the funannotate annotate function in funannotate v1.5.3 (15) from the MEROPS v12.0 (16), MIBiG v1.4 (17), Pfam v32.0 (18), dbCAN v7, and eggNOG v4.5.1 (19) databases. Transmembrane and secreted proteins were annotated using Phobius v1.0.1 (20) and SignalP v4.1 (21). Finally, secondary metabolite biosynthetic gene clusters were identified with antiSMASH v4.2.0 (22). Default parameters were used for all software unless otherwise specified.

Table 1 shows that the five newly isolated strains exhibit a genome size range of 12.02 Mb to 20.07 Mb with an average GC content of 28.6% to 60.6%.

**TABLE 1:** Accession numbers and characteristics of kefir fungal isolates

Species	Strain	SRA accession no.	GenBank accession no.	No. of reads	Coverage (×)	Genome size (bp)	GC content (%)	No. of contigs	Contig *N*_50_ (bp)	No. of genes	Single-copy BUSCOs (%)
*Candida californica*	SB-48	SRX9449769	PUHW00000000	25,448,750	192	12,323,006	28.6	1,206	23,604	5,524	91.1^*a*^
*Candida californica*	SB-116	SRX9449771	PUHU00000000	25,749,226	194	12,320,729	28.7	981	28,622	5,490	92.0^*a*^
Kazachstania exigua	SB-178	SRX9449774	PUHR00000000	27,522,278	189	13,507,013	33.3	773	38,581	5,522	96.9^*a*^
*Kazachstania unispora*	SB-162	SRX9449773	PUHS00000000	29,185,902	225	12,020,007	32.3	432	60,809	5,464	96.8^*a*^
Rhodotorula mucilaginosa	SB-353	SRX9449775	PUHQ00000000	19,300,818	89	20,066,154	60.6	416	112,846	7,169	93.4^*b*^

a The lineage data set saccharomycetes_odb10.

b The lineage data set basidiomycota_odb10.

### Data availability.

The raw reads have been deposited at the NCBI Sequence Read Archive (SRA), and the whole-genome shotgun projects have been deposited at DDBJ/ENA/GenBank. While all these data are available under BioProject number PRJNA435582, the individual SRA and GenBank accession numbers described in this report are included in Table 1. The GenBank versions described in this paper are the first versions (01).
